# EFFICACY OF A HOME-BASED EXERCISE PROGRAM “SENIOR FIT” FOR OLDER ADULTS DURING THE COVID-19 PANDEMIC

**DOI:** 10.1093/geroni/igad104.2861

**Published:** 2023-12-21

**Authors:** Kate Hyun, Kathy Lee, Vina Lervisit, Kimberly Vanhoose, Xiangli Gu, Troyee Saha, Boni Jobaidul, Angela Liegey-Dougall

**Affiliations:** University of Texas at Arlington, Arlington, Texas, United States; University of Texas at Arlington, Arlington, Texas, United States; University of Texas at Arlington, Arlington, Texas, United States; University of Texas at Arlington, Arlington, Texas, United States; University of Texas at Arlington, Arlington, Texas, United States; University of Texas at Arlington, Arlington, Texas, United States; University of Texas at Arlington, Arlington, Texas, United States; University of Texas at Arlington, Arlington, Texas, United States

## Abstract

With elevated awareness and concerns of infectious diseases such as COVID-19, the importance of overall health and wellbeing has been highlighted. More than 25% of older adults, including individuals with underlying health conditions, did not maintain the same intensity of physical activity during the pandemic. The purpose of this study was to examine the efficacy of a home-based exercise program, Senior Fit, for older adults during the COVID-19 pandemic. Senior Fit employs conventional behavior boosting approaches to promote physical activities and reduce sedentary behaviors. Older adults (N = 58) participated in the program and completed a weekly set of exercises for eight weeks. We conducted in-depth, individual interviews with 53 participants and identified four themes related to the program: (1) positive perceptions on the structure, (2) individual variances in their overall experience, (3) perceived benefits, and (4) feedback on human interactions. The participants appreciated the structure, especially following schedules provided and using paper forms to track their activities. However, some complained the program was too easy while others thought the program was too challenging. Overall, the participants discussed physical benefits (e.g., weight loss, established exercise habits) and emotional benefits (e.g., better mood, motivations). They suggested the importance of creating an inclusive and interactive environment. Many discussed the importance of the interactions with the research team as well as other participants to stay motivated in the program during the pandemic. These findings highlighted the need to involve participant stakeholders in the development of programs to increase physical activity in older adults.

